# Comparative genomics of MRSA strains from human and canine origins reveals similar virulence gene repertoire

**DOI:** 10.1038/s41598-021-83993-5

**Published:** 2021-02-25

**Authors:** Bruno Penna, Marcella B. Silva, André E. R. Soares, Ana T. R. Vasconcelos, Mariana S. Ramundo, Fabienne A. Ferreira, Maria C. Silva-Carvalho, Viviane S. de Sousa, Renata F. Rabello, Paula T. Bandeira, Viviane S. de Souza, Paul J. Planet, Olney Vieira-da-Motta, Ana M. N. Botelho, Agnes M. S. Figueiredo

**Affiliations:** 1 Instituto Biomédico, Universidade Federal Fluminense, Niterói, Rio de Janeiro, Brazil; 2 Laboratório de Sanidade Animal, Centro de Ciências e Tecnologias Agropecuárias, Universidade Estadual do Norte Fluminense, Campos dos Goytacazes, Rio de Janeiro, Brazil; 3 Laboratório Nacional de Computação Científica, Petrópolis, Rio de Janeiro, Brazil; 4 Department of Medical Microbiology, Instituto de Microbiologia Paulo de Góes, Universidade Federal do Rio de Janeiro, Rio de Janeiro, Brazil; 5 Departamento de Microbiologia, Imunologia e Parasitologia, Universidade Federal de Santa Catarina, Florianópolis, Santa Catarina Brazil; 6 Instituto de Biofísica Carlos Chagas Filho, Universidade Federal do Rio de Janeiro, Rio de Janeiro, Brazil; 7 Perelman School of Medicine, University of Pennsylvania, Philadelphia, PA USA

**Keywords:** Antimicrobials, Bacteriology, Bacterial genetics, Clinical microbiology, Phylogeny

## Abstract

Methicillin-resistant *Staphylococcus aureus* (MRSA) is an important pathogen associated with a wide variety of infections in humans. The ability of MRSA to infect companion animals has gained increasing attention in the scientific literature. In this study, 334 dogs were screened for MRSA in two cities located in Rio de Janeiro State. The prevalence of MRSA in dogs was 2.7%. Genotyping revealed isolates from sequence types (ST) 1, 5, 30, and 239 either colonizing or infecting dogs. The genome of the canine ST5 MRSA (strain SA112) was compared with ST5 MRSA from humans—the main lineage found in Rio de Janeiro hospitals—to gain insights in the origin of this dog isolate. Phylogenetic analysis situated the canine genome and human strain CR14-035 in the same clade. Comparative genomics revealed similar virulence profiles for SA112 and CR14-035. Both genomes carry *S. aureus* genomic islands νSAα, νSAβ, and νSAγ. The virulence potential of the canine and human strains was similar in a *Caenorhabditis elegans* model. Together, these results suggest a potential of canine MRSA to infect humans and vice versa. The circulation in community settings of a MRSA lineage commonly found in hospitals is an additional challenge for public health surveillance authorities.

## Introduction

*Staphylococcus aureus* is a common human pathogen involved in a wide variety of diseases. Infections caused by this agent range from skin and soft tissue infections to life-threatening bacteremia, endocarditis, osteomyelitis, and necrotizing pneumonia^1,2^. The high incidence of human infections caused by antimicrobial-resistant bacteria, especially methicillin-resistant *S. aureus* (MRSA), has led to an increased awareness related to the presence of resistant bacteria in domestic animals. Although *S. aureus* is not the most common staphylococcal species isolated from dogs^3^, the incidence of MRSA compared with that of methicillin-susceptible *S. aureus* (MSSA) is increasing in this host^4,5^. Consequently, from the one health perspective, domestic animals may serve as MRSA reservoirs in the community.

To date, both hospital-associated MRSA (HA-MRSA) and community-associated MRSA (CA-MRSA) lineages have been identified in companion animals^6–8^. Additionally, MRSA has been isolated from livestock, a finding with clear economic and public health implications^9,10^. The risk of zoonotic transmission of MRSA between humans and companion animals has been described in households, community, and healthcare settings^11,12^. However, to our knowledge, there is no study in the scientific literature addressing phylogenetic analysis and comparative genomics between MRSA strains from human and dog origins. In the present study we detected and molecularly characterized MRSA isolates obtained from asymptomatic and infected dogs in two cities in Rio de Janeiro State, Brazil: Campos dos Goytacazes and Rio de Janeiro. The antimicrobial resistance profile and the presence of *lukSF-PV* genes (encoding Panton-Valentine leukocidin) were also assessed. Additionally, because the ST5 lineage is emerging as the predominant MRSA in Rio de Janeiro hospitals^13^, we sequenced the whole genome of human and canine isolates from the ST5 lineage to gain some insights into the origin of the canine ST5 MRSA.

## Results

### Genotyping and resistance profile of MRSA from canine origins

The overall percentage of MRSA in the dogs analyzed was 2.7%; this corresponds to five isolates from Rio de Janeiro (5/210; 2.4%) and four isolates from Campos dos Goytacazes (4/124; 3.3%). A high percentage of MRSA (56.2%; n = 9; *p* < 0.0001) was detected amongst the 16 *S. aureus* isolates collected from 334 dogs (16/334; 4.8%). The three MRSA isolates (PA68, PA69, and PA73) from nasal carriers (3/88; 3.4%) were susceptible to most of the antimicrobials tested (Table 1). It is important to highlight that among the MRSA isolates cultured from infection cases (6/246; 2.4%) some displayed multiresistance profile (Table 1). MRSA strains SA07 and SA112 were resistant to six antibiotics (ciprofloxacin, clindamycin, chloramphenicol, erythromycin, gentamicin, and tetracycline) other than β-lactams (Table 1). The MRSA positive dogs were from different households; these dogs had no contact with each other.

**Table 1:** Antimicrobial resistance and molecular characterization of the MRSA isolates collected from canines.

Strain	Specimen	Antimicrobial resistance	ST	SCC*mec*	PFGE clone	*lukSF*
SA07*	Ear exudate	CP, CL, CH, ER, G, CF, TE	5	II	NY/J	−
SA112*	Ear exudate	CP, CL, CH, ER, G, CF, TE	5	II	NY/J	−
PA68*	Nasal swab	CL, ER, CF	30	IV	USA1100	+
PA69*	Nasal swab	CF, TE	30	IV	USA1100	+
PA73*	Nasal swab	CL, ER, CF	30	IV	USA1100	+
NF62**	Ear exudate	CF, ER	1	IV	USA400	+
NF191**	Exudate from soft tissue tumor	CF	30	IV	USA1100	+
NF601**	Exudate from surgical site infection	CL, CH, ER, TE	239	III	BEC	−
NF661**	Skin exudate	CL, CH, ER, TE	239	III	BEC	−

*CP* ciprofloxacin, *CL* clindamycin, *CH* chloramphenicol, *ER* erythromycin, *G* gentamicin, *CF* cefoxitin, *TE* tetracycline, *ST* MLST sequence typing, *NY/J* New York/Japan clone, *BEC* Brazilian epidemic clone, + presence, − absence, *Rio de Janeiro, **Campos de Goytacazes.

**Figure 1:**
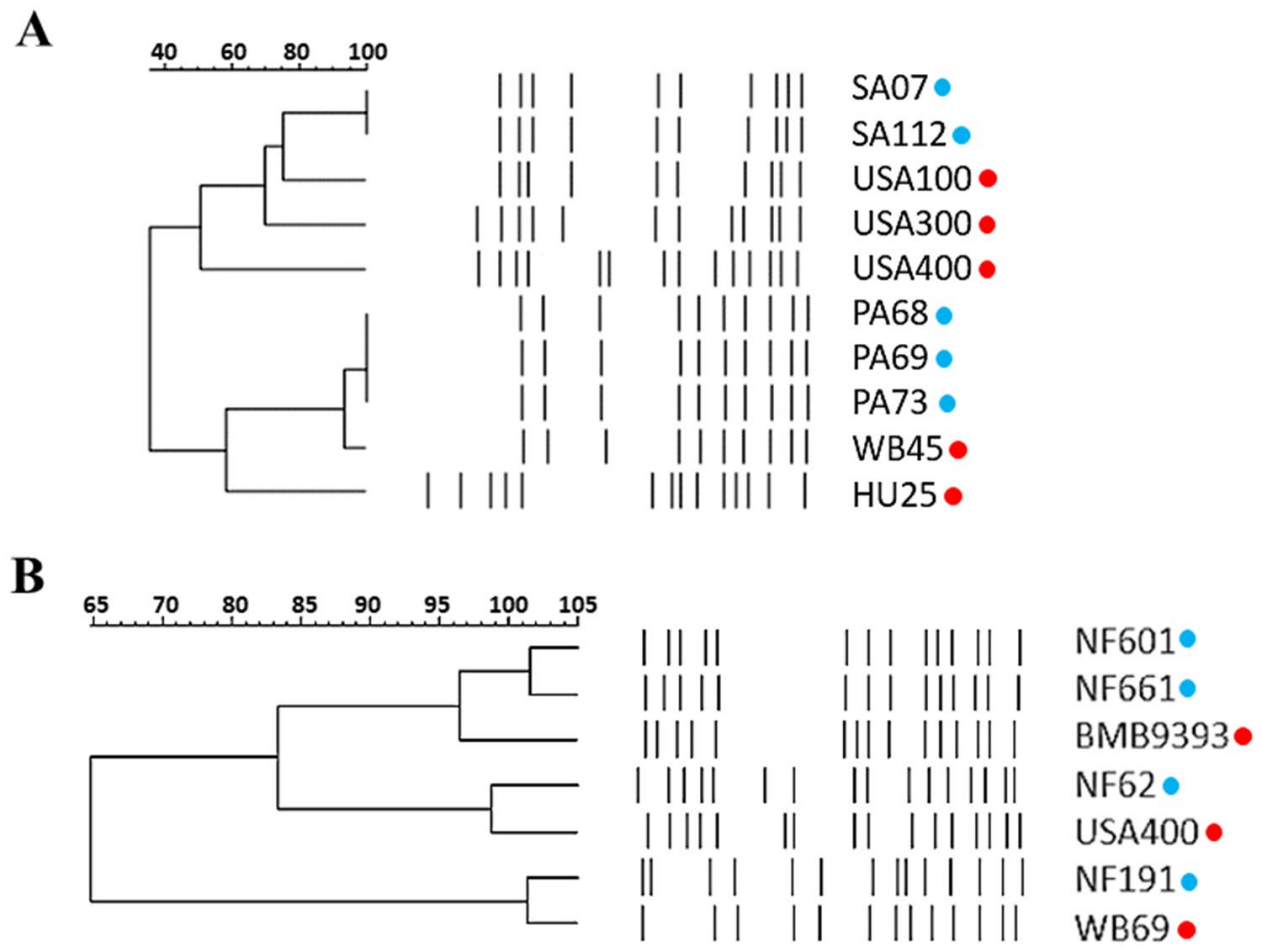
Pulsed-field gel electrophoresis (PFGE) of the *Sma*I-fragmented genomic DNA of MRSA isolates recovered from healthy and infected dogs in the state of Rio de Janeiro (red circles). (**A**) Dog isolates from Rio de Janeiro city and (**B**) dog isolates from Campos de Goytacazes city. USA100, USA300, USA400, WB45 and WB69 (USA1100), and BMB9393 and HU25 (BEC) were representatives of the MRSA international clones used for comparison purposes (blue circle). Dendrograms were generated from the PFGE patterns using GelCompar II software version 6.5.

The isolates PA68, PA69, and PA73 (healthy dogs) and NF191 (infected soft tissue tumor) carried the SCC*mec* type IV, *lukSF-PV* genes, and displayed a PFGE pattern that resembled the strains WB45 (PA68, PA69, PA73) and WB69 (NF191), both of which were previously typed as PVL + and belong to the ST30-SCC*mec* IV linage, related to the Oceania Southwest Pacific Clone (OSPC/USA1100; Fig. 1). In addition, these USA1100-related isolates from dogs displayed the MLST allelic profile (2-2-2-2-6-3-2) corresponding to ST30. Two MRSA isolates (SA07 and SA112; both from ear exudates) carried SCC*mec* II. They did not harbor *lukSF-PV* genes and displayed a PFGE quite similar to that of HA-MRSA from the New York/Japan clone, also called USA100, which belongs to the ST5-SCC*mec* II lineage (Fig. 1A). In fact, these two canine SCC*mec* II isolates were typed as ST5. According to the dendrogram (Fig. 1B), the PFGE banding pattern of the MRSA isolate NF62 (collected from ear exudate) had 93.75% similarity with the band pattern of the strain USA400-0051, a representative of the USA400 clone. These data were further confirmed by MLST (allelic profile: 1-1-1-1-1-1-1), which allocated NF62 to ST1, and by SCC*mec* typing, which classified this isolate as SCC*mec* IV. In addition, the *lukSF*-*PV* genes were detected in NF62. Finally, the isolates NF601 (surgical site infection) and NF661 (pyoderma case) according to PFGE analysis were related to the strain BMB9393 (Fig. 1B); a representative of the Brazilian epidemic clone (BEC; ST239-SCC*mec* III). These canine isolates also carried SCC*mec* III and ST239 allelic profile (2-3-1-1-4-4-3), confirming the clonal association with BEC (Table 1).

### Genomic comparison between canine and human CC5 MRSA

**Figure 2:**
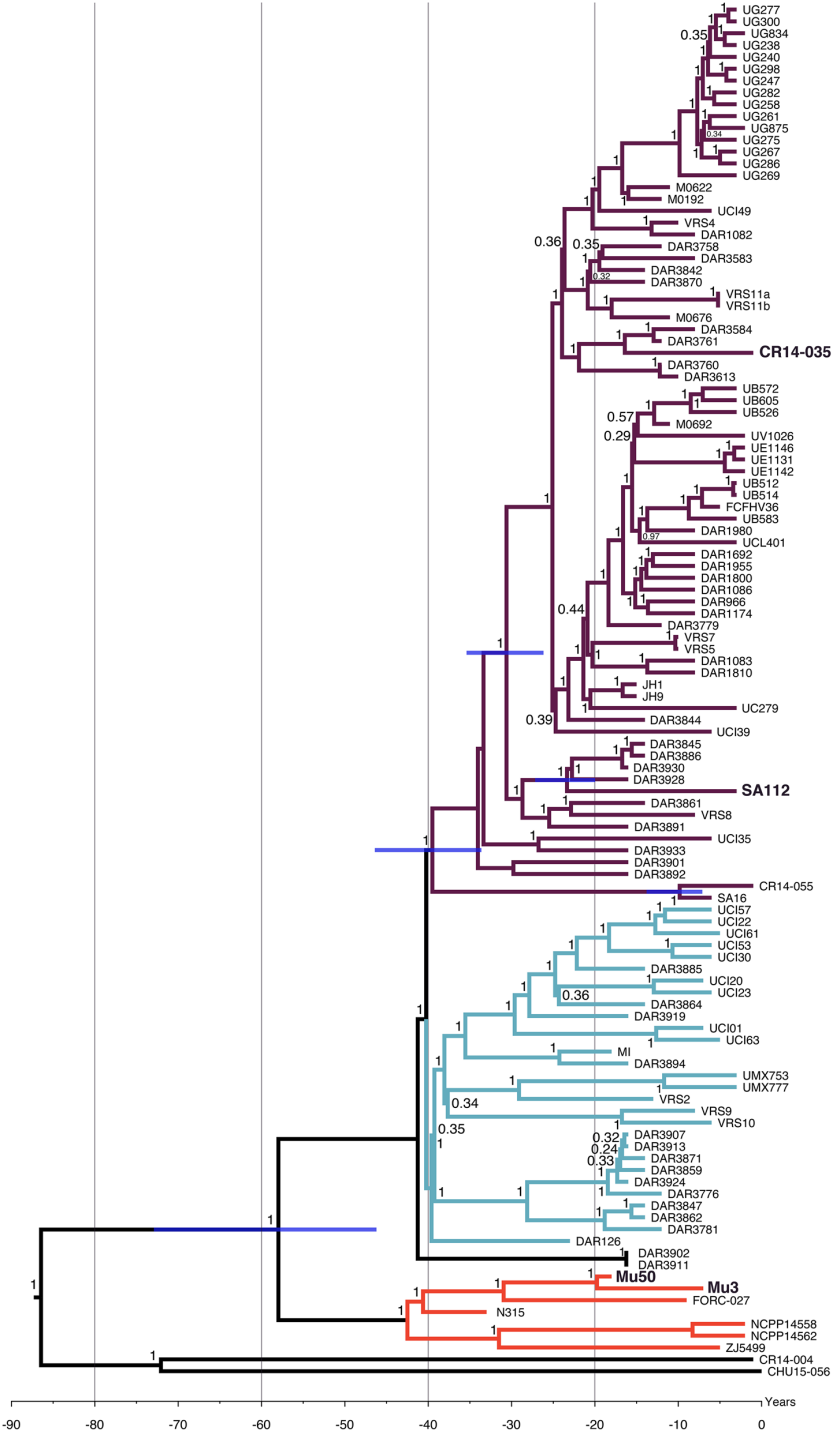
A time-calibrated Bayesian phylogeny for MRSA strains of the lineage ST5-SCC*mec* II from canine (SA112) and human origins obtained from 116 core genome alignments. Values at nodes indicate the posterior probabilities for each node. The tree is drawn to a time-scale indicated in years. Blue bars indicate the 95% credibility intervals (CI) for some node ages. The 95% CI for all nodes can be seen at Supplementary Figure S1. The year of 2015 was used as a reference point for chronological estimates, which corresponds to the isolation year of the most recent strain used for the tree construction.

The general genomic characteristics of the MRSA of the ST5-SCC*mec* II lineage from a canine (strain SA112) and humans (strains CR14-035, CR14-004, CR14-055, and CHU15-056), used for whole genome sequencing, are presented in Table 2. The Bayesian phylogenetic inference using these and other CC5 genomes publicly available in GenBank revealed that the canine strain SA112 grouped in a clade (purple), which includes genomes of human-derived strains (Fig. 2). This clade grouped mostly the genomes of strains from the American continent (e.g.: USA, Brazil, Colombia, Chile, Mexico, Guatemala, among other countries) including CR14-035 and CR14-055 from hospitalized patients in Rio de Janeiro, and another Brazilian strain (FCFHV36) previously isolated from a case human osteomyelitis in the state of Santa Catarina, in the southern region of Brazil^14^. In addition, the purple clade clustered the genomes of the strains JH1 and JH9 collected from blood of a patient with congenital heart disease and endocarditis who was treated extensively with vancomycin without success in Baltimore, Maryland, USA (Fig. 2). The blue clade also grouped CC5 genomes from humans mostly from USA and the red clade from Asia; this last clade includes the genomes of Mu50 and Mu3 strains from Japan (Supplementary Table S2). It was also estimated that the canine SA112 strain shared a common ancestor with human MRSA strains from USA (e.g.: DAR3928, DAR3939, DAR3845, DAR3886) at 20.54 years before 2015 (19.97–21.17 95% HPD). The divergence of SA112 and CH14-035 (human strain from Rio de Janeiro) was estimated at 30.61 years before 2015 (26.17–35.4 95% HPD). However, SA112 and the human strains CH14-004 and CH15-056 from Rio de Janeiro might have diverged at more than 85 years before 2015. Given this older inferred date, the acquisition of *mecA* gene by these two human and animal strains seems more likely to have occurred as distinct genetic events in different MSSA strains. It is interesting that the archetype strain Mu50, a vancomycin-intermediate resistant *S. aureus* (VISA), isolated from a baby with a surgical site infection in Japan^15^, was grouped in an independent clade (red) with Mu3^16^ and N315^15^, both MRSA strains isolated from patients in Japan. MU50 and Mu3 shared an ancestor at 20.04 years before 2015 (18.85–21.52 95% HPD). Therefore, in this prediction, Mu50 and Mu3 strains have emerged in Japan by 1996–1994 and the first report of these strains is dated 1996^16^, in agreement with the calibration of the chronology calculated in our study. It was estimated that the genomes clustered in the red clade (Asia) diverged from those grouped in the blue and purple clades (America) approximately at 1957 (1967–1942%HPD) (Fig. 2).

**Table 2:** Characteristics of the completely closed genomes from ST5 MRSA strains sequenced in this study.

Isolate	Host	Specimen	Isolation year	GenBank accession number	Chromosome size (bp)	GC content (%)
SA112	Dog	Ear exudate	2012	CP020553	2,982,283	32.9
CR14-035	Human	Nasal swab	2014	CP020544	2,989,067	32.9
CR14-004	Human	Tracheal secretion	2014	CP021105	2,745,711	32.9
CR14-055	Human	Blood	2014	CP021057	2,854,872	32.9
CHU15-056	Human	Blood	2015	CP021171	2,773,467	32.9

The strain SA112 is from canine origin and all others from humans.

Information about genome assembly and BioProject can be obtained by accessing the GenBank.

**Figure 3:**
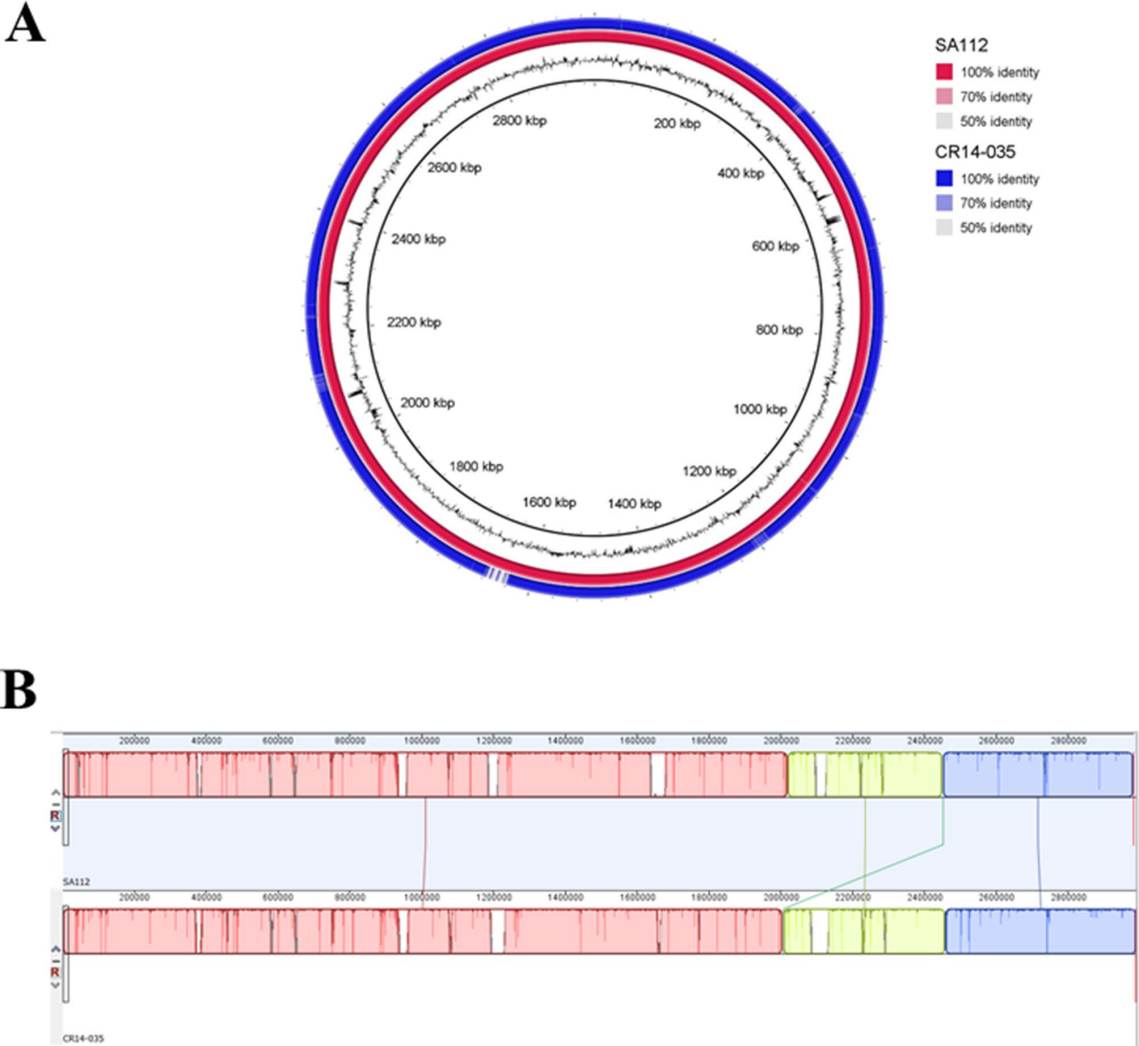
Genome alignments using blast ring generator (BRIG) and multiple alignment of conserved genomic sequence (MAUVE). (**A**) Overview of the completely closed genomes of the analyzed ST5 MRSA using BRIG. The color circles represent the genomes of each sequenced strain. The outer black circle represents the GC content and the inner the reference genome of the archetypal CA-MRSA strain MW2. Circular maps of ST5 genomes obtained from ST5 MRSA strains collected from canines (SA112—red circle) and humans (CR14-035—blue circle). The reference genome was the genome of the SA112. (**B**) Alignment between whole genomes of ST5 MRSA strains SA112 (from canine) and CR14-035 (from human) using MAUVE. Note that only four local collinear blocks were identified, with a translocation of the smallest block (represented by the green line).

**Figure 4:**
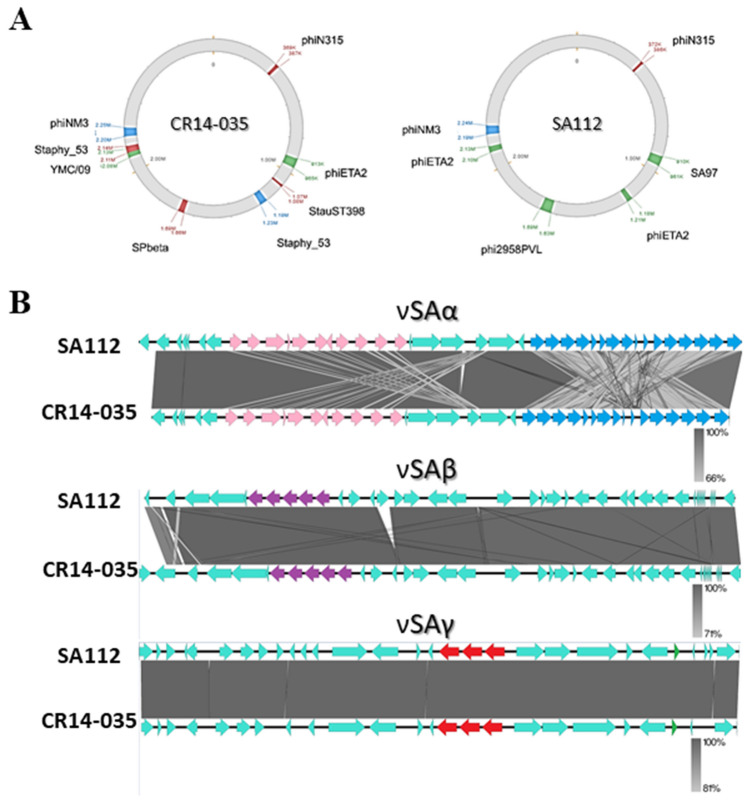
Regions of genomic plasticity corresponding to bacteriophages and important genomic islands. (**A**) Schematic representation of the position of bacteriophages in the ST5 genomes of the MRSA strains SA112 (from canine) and CR14-035 (from human) sequenced in this study. (**B**) Alignment of the genomic island carrying important virulence genes in the genome of the strains SA112 (from canine) and CR14-035 (from human). In νSAα the *set* genes are highlighted in pink and *lpl* genes are highlighted in blue. The *splABCDF* operon in νSAβ is highlighted in purple. In νSAγ, *set* genes are highlighted in red and *psmβ* is highlighted in green.

Based on the phylogenetic analysis, the completely closed genome of the human strain CR14-035 (ST5-SCC*mec*II) from Rio de Janeiro was chosen for the comparative genomic analysis with the genome of the canine strain SA112 (ST5-SCC*mec*II). Whole genome alignment revealed 95.36% of nucleotide identity between both genomes. The analysis of the circular genomes by BLAST Ring Image Generator (BRIG; Fig. 3A) and the chromosomal architecture using MauveProgressive alignment (Fig. 3B) confirmed the high nucleotide conservation. A total of four local co-linear blocks were obtained for these two genomes (Fig. 3B). The few synteny breaks observed were mostly associated with differences in phage profiles. A bacteriophage search revealed differences not only in the type and quantity of phages but also in the position of these phages on the bacterial chromosome, as shown in Fig. 4A. Only one bacteriophage, related to phiN315, was found in both genomes, in a similar position. Despite the differences between the human and canine MRSA genomes analyzed, all bacteriophages found in the SA112 genome have also been found in the genomes of other *S. aureus* strains of human origin as indicated in the BLAST searches.

The isolate SA112 showed phenotypic resistance to ciprofloxacin, clindamycin, chloramphenicol, erythromycin, gentamycin, cefoxitin, and tetracycline (Table 1). Resfinder analysis detected genes encoding resistance to lincosamides (*ermA*), aminoglycosides (*aadD*) and beta-lactams (*mecA*) and found mutations on *gyrA* (S84L) and *grlA* (S80F) that leads to the phenotypic resistance to ciprofloxacin. No genes encoding resistance for chloramphenicol and tetracycline could be detected.

Both genomes carry the genomic islands νSAα, νSAβ, and νSAγ, for which the primary virulence associated genes are listed in Table 3 (Fig. 4B). Unlike other CC5 strains, such as Mu50, which carries staphylococcal pathogenicity island (SaPI), SA112 and CR14-035 genomes do not carry SaPI1, SaPI2, or SaPI3—which often harbor genes associated with toxic shock syndrome-TSST-1 (*tst*, *seb*, and *sec* genes).

**Table 3:** Genomic islands detected in the completely closed genomes sequenced in this study obtained from ST5 MRSA strains collected from humans (CR14-035) and canine (SA112).

Island	Reference	Main target	SA112	CR14-035
νSAα	Mu50	*set, lpl*	+	+
νSAβ	Mu50	*splABCDF*	+	+
νSAγ	Mu50	*set, psmβ*	+	+
SaPI1	COL	*seb, tst, ear*	−	−
SaPI2	Mu50	*sel, sec, tst*	−	−
SaPI3	Mu50	*sel, sec, ear*	−	−

+ presence, − absence.

Additional analysis of the virulence gene repertoire between the canine and human genomes from Rio de Janeiro showed that many enterotoxins or enterotoxin-like encoding genes including *seg*, *sei, sem, sen*, and *seo* were detected. Some representative human genomes grouped in the purple, blue, and red clades (MU50, JH1, JH9, FCFHV36, CR14-035, CR14-055, and MI) were also included in this analysis. Not only were absent the genes *sec*, *sel* and *tst* in the MRSA genomes analyzed from humans but also they were equally missed in the CC5 genome from dog, SA112 (Supplementary Table S1). All genomes carried genes related to biofilm formation, such as the *ica* operon, the autolysin gene (*atl),* several exoproteins, and genes related to immune evasion (Supplementary Table S1). Notably, all virulence genes of the strain SA112 located in the island regions analyzed displayed high level of nucleotide identity (100%) with the correspondent genes carried by the MRSA strains of human origins; especially by those from Rio de Janeiro State (Supplementary Table S1). Exceptions were only observed for *icaB*, *sak* and *scn* for which all MRSA strains from Rio de Janeiro (dog and human origins) showed 99.89%, 99.39% and 99.72% of nucleotide identity, respectively (Supplementary Table S1).

### Virulence assessment

**Figure 5:**
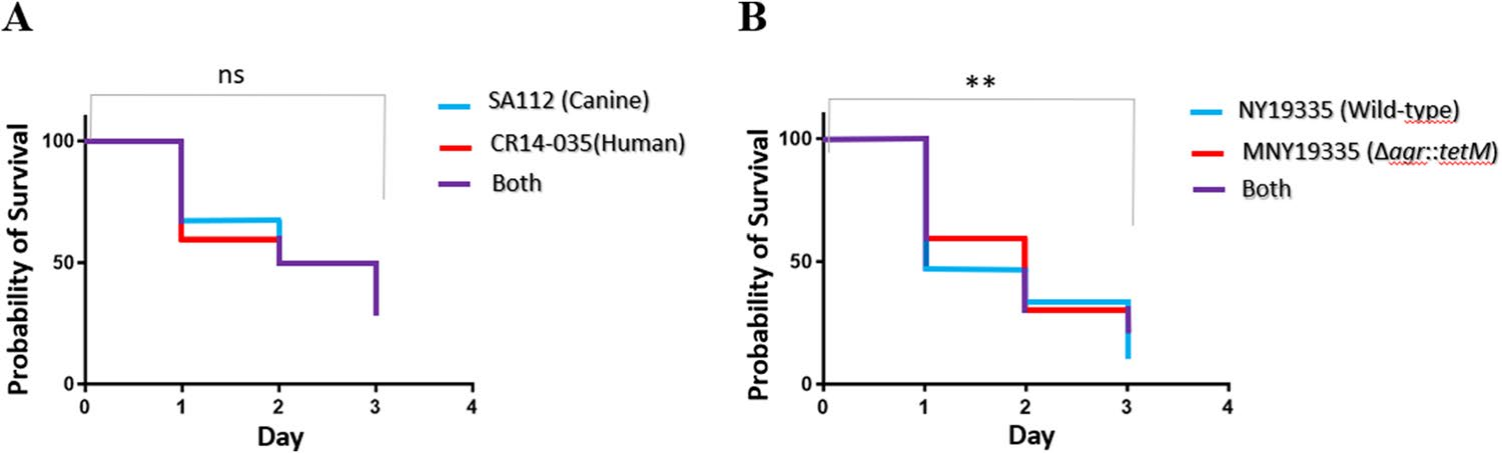
Comparison of survival curves of *C. elegans* to assess the virulence of the isolates. (**A**) The nematodes were separately infected with strain SA112, obtained from a dog and CR14-035, obtained from humans**.** (**B**) No significant difference was found. The set of strainsNY19335—an Agr-functional CC5 clinical isolate—and MNY19335∆*agr*::*tetM*—the isogenic *agr* knockout mutant—was used to control the experiments. *Ns* not significant; **p < 0.01.

*Caenorhabditis elegans* model was used to compare the virulence potential of the canine-derived CC5 MRSA (SA112) with that of human MRSA (CR14-035). No difference was found between the killing curves of the CC5 strains with human and animal origins (31.4% and 29.1% of worm survival on the 3rd day of the experiment, respectively), as shown in Fig. 5A. Because *agr*-knockout mutants display significant reduction in their virulence potential^17–19^, we used a positive control Agr-functional *S. aureus* wild-type strain (NY19335) and its isogenic knockout mutant (MNY19335; Δ*agr*::*tetM*) as a negative control, to validate this model for *S. aureus*. As expected, a difference was detected in the survival curves for the *agr*-functional strain and the isogenic knockout mutant (10.4% and 22.9% of worm survival on the 3rd day of the experiment, respectively), as shown in Fig. 5B.

## Discussion

Methicillin resistance has been increasingly reported in staphylococcal isolates from canines in several countries^20–24^. The overall rate of MRSA among the 334 dogs in this study (2.7%) was similar to that observed elsewhere (varying from 1 to 6%) in locations such as Korea (0.6%), Japan (1%), Australia (4%) and the United States (5.7%)^6,20–22^. In our study, the prevalence of nasal colonization by MRSA in dogs (3.4%) was similar to that reported in this country for healthy human carriers (2.3%)^25^. However, it is of note that 56.2% of *S. aureus* isolates from the dogs were MRSA. Such high rates have also been observed in other countries—62.7% in Germany^23^ and 51.8% in the United States^24^. Although *S. aureus* is not the most common species isolated from canine infections, it is of concern that MRSA is becoming more prevalent than MSSA in dogs in some regions. The presence of MRSA in dogs has important implications for public health because of the high level of exposure between housed dogs and humans^24^. Studies have already suggested the possible transmission of *S. aureus* strains among dogs and their owners^26,27^. However, only a few studies have addressed this question using genomic strategies^22,28,29^.

The MRSA isolates from dogs detected in this study belonged to lineages commonly found infecting humans in Brazil and other countries^2,30,31^. The PVL-producer isolates from dogs were related to the CA-MRSA clones USA400 (NF62) or USA1100 (PA68, PA69, PA73). Of note, all USA400-related isolates previously detected from humans in Brazil have been associated with healthcare-associated infections and were PVL negative^30^. Although not as frequent as the USA300 clone, the USA400 clone is the second most common PVL-positive CA-MRSA associated with human community-acquired infections in North America^1^. The epidemiological data obtained relative to the canine isolate NF62 strongly suggest that the dog had acquired this PVL-positive USA400 isolate in the United States, a country where the animal had temporarily stayed prior to sample collection in Brazil, suggesting the potential that pets have for introducing new CA-MRSA clones in distant places.

The USA1100 CA-MRSA found in asymptomatic nasal carriers and infected dogs has previously been reported in Brazil in human infections in Rio de Janeiro^29^. Additionally, it was detected in the nasal cavity of a healthy cat in Rio de Janeiro^7^ and a healthy dog in Japan^20^. BEC-related isolates were the predominant MRSA in Rio de Janeiro hospitals from 1995 to 2009; they do not produce PVL and are typically human-associated hospital isolates^32–34^. As most BEC isolates from humans, the ST239-SCC*mec* III isolates recovered from diseased dogs (NF601 and NF661) are multidrug resistant.

The USA100-related MRSA recovered from the ear exudates of infected dogs is emerging as the predominant MRSA associated with human infections in Rio de Janeiro hospitals, replacing ST239^13,35^. Even though the overall incidence of MRSA in canines is still low in the cities analyzed—roughly 2–3 animals out of 100 will carry MRSA—some will be infected by these bacteria and may be untreatable with currently recommended drugs to treat companion animals. A research performed to access the risk factors for MRSA infections in dogs and cats reported as significant influences (1) the number of antimicrobial courses, (2) admission days to veterinary clinics, and (3) submission to surgical implants. In addition, the odds of contact with humans which had been hospitalized were higher in MRSA infected pets when compared with MSSA controls^36^.

Our Bayesian phylogenetic inference agrees with the previous dating done by Challagundla et al.^37^, although different clocks were used. Our purple clade, that grouped the animal strain SA112, and also our human strains CR14-035 and CR14-055, corresponded with the clade named CC5-II B by Challagundla et al. Also our blue clade corresponded with CC5-II-A and our red clade, that clustered the strains N315, Mu50 and Mu3 among others, matched the CC5-Basal clade of Challagundla et al.

It is conceivable that increased number of companion animals have intensified the exchange of bacteria between humans and their pets^22,38,39^. Worthing et al.^22^ analyzing MRSA isolates from Australia found that animal and veterinarian-derived MRSA were also intermingled on the phylogenetic tree. Likewise, Harrison et al.^38^ showed that ST22 MRSA isolates (EMRSA-15 clone) from cats and dogs in the United Kingdom were interspersed with human isolates throughout the epidemic EMRSA-15 pandemic clade. In many cases, human isolates were basal to those from companion animals, suggesting a human source for isolates infecting companion animals^38,40^.

It has been suggested that after transmission to a new host a pathogen may evolve and adapt by losing and/or acquiring new MGEs^41,42^. Loeffler et al.^28^ observed significant differences in MGE content between pets’ and their owners’ isolates. These authors concluded that the variation found amongst MGEs highlights the genetic adaptation of MRSA in different hosts^28^. In our study, the human and canine strains shared most MGEs and, despite the differences found in phage profiles, all phages detected in the animal strain had already been detected in human *S. aureus* isolates. Additionally, besides humans and canine genomes sharing a conserved set of virulence genes and comparable virulence potential in a *C. elegans* model, the canine genome was allocated between human genomes in the same phylogenetic clade. The *C. elegans* survival model used here was able to distinguish levels of virulence between the *agr*-null mutant and its isogenic wild-type validating this model for *S. aureus*.

Taken together, these results suggest that the canine ST5 strain may represent a human-to-animal transmission. The incidence of MRSA in the cities studied is still relatively low. In consequence, only one CC5 genome from a dog was analyzed. Therefore, our data should be analyzed considering such limitation. There have also been reports of isolation of USA100 (ST5-SCC*mec* II MRSA) from dogs and other domestic animals in the United States^4,27,43,44^. This finding is concerning because CC5 isolates can display high level of antibiotic multiresistance. Although still rare, VISA and VRSA resistances have mostly been described among CC5 strains^45–47^.

## Conclusions

This work demonstrates that MRSA isolates were detected among infected and colonized dogs in the state of Rio de Janeiro. The isolates recovered from those animals are related to the CA-MRSA international clones USA1100/OSPC (ST30-SCC*mec* IV) and USA400 (ST1-SCC*mec* IV) and to the HA-MRSA clones USA100/NY/Japan (ST5-SCC*mec* II) and BEC ST239-SCC*mec* III) that are commonly involved in human infections in Brazil and in other countries. Because ST30 isolates are the primary CA-MRSA pathogens in Brazil, the presence of this PVL-producing MRSA in animals in two cities in Rio de Janeiro state is of great concern. Additionally, a high rate of methicillin resistance was found among the *S. aureus* detected, and two MRSA isolates were resistant to a wide variety of other antibiotics. We have demonstrated a high conservation of virulence traits between human and canine MRSA genomes. Indeed, the canine genome interspersed with human genomes of MRSA in the phylogenetic tree suggesting a possible human origin for the canine ST5 strain and raising the possibility that this strain can move easily between hosts without canine-specific adaptations. The circulation of pandemic lineages of MRSA, commonly found in hospitals and community settings is an additional challenge for public health authorities. Monitoring and surveillance of companion animals for MRSA would be beneficial to better define the circulation of these resistant strains and to assess the potential risk of animals as sources of resistant infections in humans.

## Methods

### Bacterial isolates

Three hundred and thirty-four dogs were screened for MRSA in a prospective study carried out between 2010 and 2013 in Rio de Janeiro (n = 210) and Campos dos Goytacazes (n = 124) cities, located in Rio de Janeiro state, 280 km apart. The dogs were male and female adults (age: 1–8 years) and either healthy (n = 88 nasal swabs) or infected animals (swabs of ear secretions and skin exudates; n = 246). The clinical material was collected from dogs brought to the Department of Small Animal Practice of the Veterinary Hospital at Universidade Estadual do Norte Fluminense (UENF), located in Campos dos Goytacazes, Rio de Janeiro, Brazil. Also, from dogs who visited a number of private veterinary clinics in Rio de Janeiro city. All of the samples were collected using sterile cotton swabs (Copan Diagnostic, Italy). Only one MRSA isolate from each dog was studied. This study was approved by the ethics committee of Federal Fluminense University (#218/10) and by the Ethics Committee for Animal Care and Use from UENF Darcy Ribeiro (#145/2011). The swabs were inoculated in tryptic soy agar (TSA; Difco, Franklin Lakes, NJ, USA) and mannitol salt agar (MSA; Merck, Darmstadt, Germany). After the samples were incubated at 37 °C/18 h, we examined cell morphology using the Gram-staining method. Gram-positive colonies were tested for catalase and free coagulase production. *S. aureus* isolates were identified using a polymerase chain reaction (PCR) *nuc*-based test described by Sasaki et al. (2010)^48^. The MRSA strains USA100-0022 (USA100), USA300-0114 (USA300), USA400-0051 (USA400), WB45 and WB49 (USA1100), BMB9393 and HU25 (BEC) were the representatives of MRSA international clones used to compare PFGE band differences.

### Antimicrobial susceptibility

Resistance to methicillin was detected using 30-μg cefoxitin disks^49^. All of the MRSA isolates were also tested for susceptibility to ciprofloxacin (5 μg), clindamycin (2 μg), chloramphenicol (30 μg), erythromycin (15 μg), gentamicin (10 μg), rifampicin (5 μg), tetracycline (30 μg), and sulfamethoxazole + trimethoprim (23.75/1.25 μg) using a disk diffusion test, as recommended by the Clinical and Laboratory Standards Institute (CLSI)^49^. The disks were obtained from Cecon (São Paulo, SP, Brazil), and the *S. aureus* strain ATCC 25923 was used as a control.

### Molecular characterization

The presence of the *mecA* gene in cefoxitin-resistant *S. aureus* was confirmed by PCR^50^. Methicillin-resistant *S. aureus* genotyping was performed using several methods including SCC*mec* typing^51^, restriction and modification (RM) testing^52,53^, pulsed-field gel electrophoresis (PFGE) of the *Sma*I-fragmented DNA^54^, and multilocus sequence typing (MLST)^55^. Dendrograms were generated from the PFGE patterns using GelCompar II software 6.5 (Applied Maths, Sint-Martens-Latem, Belgium). To assign the MLST sequence types, the allele sequences were trimmed and analyzed using the Public Database for Molecular Typing and Microbial Genome Diversity (http://pubmlst.org). Detection of *lukSF-PV* genes was performed using a PCR-based method described previously^56^.

### Whole-genome sequencing

Because some MRSA isolates from dogs belonged to the ST5-SCC*mec* II lineage, which has recently emerged as the predominant lineage in Rio de Janeiro hospitals^13^, we aimed to investigate whether these canine ST5 isolates were from a human origin or were specific to animals. To achieve this goal, we performed whole genome sequencing of five ST5-SCC*mec* II isolates from humans and canines (Table 2). Genomic DNA was prepared from overnight cultures using the Wizard Genomic DNA Purification Kit (Promega Corporation, Madison, WI, USA). The bacterial cells were centrifuged, washed with TSM buffer (50 mM Tris, pH 7.5; 0.5 M sucrose; 10 mM MgCl_2_), lysed with 50 U/mL lysostaphin (Sigma-Aldrich, Merck, Darmstadt, Germany), and processed using the protocol suggested by the manufacturer. Genome libraries were prepared using the Nextera XT kit (Illumina, San Diego, CA, USA) and sequenced using the Illumina MiSeq instrument (paired end reads of 125 bp). Genome assembly was performed by combining de novo and map to reference assembly methods, through Velvet v7.0.4^57^ and Geneious v11.0.5^58^, respectively. The genomes were submitted to the NCBI Prokaryotic Genome Annotation Pipeline^59^.

### Phylogenetic analysis

For this analysis, besides the five ST5-SCC*mec* II genomes sequenced in this work, we included 111 CC5-SCC*mec*II genomes publicly available in the GenBank (Supplementary Table S2). We ran core genome alignments with REALPHY^60^ and used ClonalFrameML^61^ to further verify the absence of recombination events that could possibly affect our molecular dating results. The phylogenetic tree under a Bayesian framework was obtained using the package BEAST v1.10.4^62^, assuming a GTR + Γ nucleotide substitution model^63,64^. The tree was reconstructed using an uncorrelated log-normal relaxed molecular clock model^65^, with a random start tree and a coalescent constant size tree prior^66^. We used for the substitution rate prior a value of 1.5 × 10^–6^ as ucld.mean parameter (mean of the log-normal distribution), allowing the distribution to vary between 1.0 × 10^–10^ and 1.0 × 10^–3^. The final substitution rate inferred in this analysis was 2.00 × 10^–6^ substitutions per site year^−1^ (with 95% HPD interval of 1.62 × 10^–6^ and 2.40 × 10^–6^). This inferred rate is similar to that obtained by Duchêne et al.^67^. We calibrated the molecular clock using tip dates (sampling times) for all individuals. Nine independent MCMC chains for 200 million iterations each were run, combining them after discarding the first 10% as burn-in. We visualized the MCMC chains using Tracer v1.6^68^ calculated the maximum clade credibility tree using TreeAnnotator v2.5^69^ and edited the final tree using FigTree v1.4.3^70^. The year of 2015 was used as a reference point for chronological estimates, which corresponds to the isolation year of the most recent strain used for tree constructions.

### Genomic analysis

For most of the comparative genomics analysis, we selected the genome of the human strain CR14-035 from Rio de Janeiro city; the most closely related to that of the canine strain SA112 based on information in the phylogenetic tree. BLAST Ring Image Generator 0.95^71^ was used to display circular comparisons between the genomes, using the canine genome as reference. We analyzed chromosomal architecture and genome organization using the progressive Mauve genome alignment algorithm^72^, with default parameters. Bacteriophage analysis was conducted using Phage Search Tool Enhanced Release (PHASTER)^73^. Resfinder^74,75^ was used to identify the resistance genes in SA112 genome. The staphylococcal pathogenicity island (SaPI) and other genomic islands (GIs) were annotated with the support of Island Viewer^76^. We also relied on manual annotation using the scientific literature. Well known virulence genes from *S. aureus* were searched using Local BLAST^77^ not only in SA112 and CR14-035, but also in all other CC5 genomes included in the phylogenetic analysis. The genomes were considered positive for each gene applying a cut-off of 90% coverage and 96% identity using gene sequences from N315 (Genbank Accession number: BA000018).

### Virulence potential assessment

The nematode *Caenorhabditis elegans* is increasingly recognized as relevant for the study of bacterial pathogenesis. This model is susceptible to different human pathogens that are able to infect the intestine of the nematode. Various genes related to mammalian immune response are not encoded in the nematode genome. However, *C. elegans* has an immune response that utilizes a number of evolutionarily conserved signaling pathways, including a highly conserved mitogen-activated protein kinase (MAPK) signaling pathways that activates the innate immune response to bacterial infections^78^. This system is easy to manipulate, has a good reproducibility and is not under subject of ethical consideration. Because of that, it is considered an interesting model to study virulence and other aspect of host-bacteria interactions. We used the *C. elegans* survival assay to compare the virulence potential of the strains CR14-035 and SA112 from humans and dogs, respectively, based on a model described previously in the literature^79^, with some modifications. Briefly, to produce the bacterial lawns, a single colony was inoculated in trypticase soy broth (TSB; BD) and the culture was incubated at 37 °C for 4 h with shaking (350 rpm). Then, a 10-μL aliquot of the culture was homogeneously spread to form a bacterial lawn on the wells of 96-well microtiter plates containing TSA supplemented with 5 μg/mL nalidixic acid and 5 μg/mL of cholesterol. For each assay, about 5–20 L4-stage nematodes were transferred to the wells containing lawns formed by each of the tested isolates. The plates were incubated at 25 °C and monitored every 24 h for live and dead worms over the course of 3 days. Each MRSA isolate was tested in three completely independent experiments with 12 replicates each and the data shown are an average of these experiments. Because *agr*-knockout mutants are less virulent than wild-type strains with functional *agr* systems in different in vivo and ex vivo models^17–19^, we carried out a control using the wild-type strain NY19335 (ST5-SCC*mec* II MRSA, isolated from bacteremia) and the isogenic *agr* knockout MNY19335 (∆*agr*::*tetM*)^17^ to assess the accuracy of this *C. elegans* model for distinguishing virulence potential in *S. aureus* human pathogen.

### Statistical analysis

Nematode survival rates were calculated using the Kaplan–Meier method and tested for significance using the log rank test (Software, Inc., La Jolla, CA, USA). In addition, we used the chi-square test to calculate the significance of the increased rate of MRSA among the animals*. p* values less than 0.05 were considered to be statistically significant.

### Guidelines statements

All methods were carried out in accordance with relevant guidelines and regulations. The study was carried out in compliance with the ARRIVE guidelines.

## Supplementary information

Supplementary figure S1.

Supplementary table S1.

Supplementary table S2.

## References

[1] David MZ, Daum RS (2010). Community-associated methicillin-resistant *Staphylococcus aureus*: Epidemiology and clinical consequences of an emerging epidemic. Clin. Microbiol. Rev..

[2] Pardos de la Gandara M (2016). MRSA causing infections in hospitals in greater metropolitan New York: Major shift in the dominant clonal type between 1996 and 2014. PLoS One.

[3] Penna B (2010). Species distribution and antimicrobial susceptibility of staphylococci isolated from canine otitis externa. Vet. Dermatol..

[4] Lin Y (2011). Evidence of multiple virulence subtypes in nosocomial and community-associated MRSA genotypes in companion animals from the upper midwestern and northeastern United States. Clin. Med. Res..

[5] van Balen J (2013). Presence, distribution, and molecular epidemiology of methicillin-resistant *Staphylococcus aureus* in a small animal teaching hospital: A year-long active surveillance targeting dogs and their environment. Vector Borne Zoonot. Dis..

[6] Hoet AE (2013). Epidemiological profiling of methicillin-resistant *Staphylococcus aureus*-positive dogs arriving at a veterinary teaching hospital. Vector Borne Zoonot. Dis..

[7] Quitoco IMZ (2013). First report in South America of companion animal colonization by the USA1100 clone of community-acquired meticillin-resistant *Staphylococcus aureus* (ST30) and by the European clone of methicillin-resistant *Staphylococcus pseudintermedius* (ST71). BMC Res. Notes.

[8] Davis JA (2014). Carriage of methicillin-resistant staphylococci by healthy companion animals in the US. Lett. Appl. Microbiol..

[9] van Duijkeren E (2010). Methicillin-resistant *Staphylococcus aureus* in horses and horse personnel: An investigation of several outbreaks. Vet. Microbiol..

[10] Feingold BJ (2012). Livestock density as risk factor for livestock-associated methicillin-resistant *Staphylococcus aureus*, the Netherlands. Emerg. Infect. Dis..

[11] Manian FA (2003). Asymptomatic nasal carriage of mupirocin-resistant, methicillin-resistant *Staphylococcus aureus* (MRSA) in a pet dog associated with MRSA infection in household contacts. Clin. Infect. Dis..

[12] Nienhoff U (2009). Transmission of methicillin-resistant *Staphylococcus aureus* strains between humans and dogs: Two case reports. J. Antimicrob. Chemother..

[13] Chamon RC, Ribeiro S, Da S, da Costa TM, Nouér SA, DosSantos KRN (2017). Complete substitution of the Brazilian endemic clone by other methicillin-resistant *Staphylococcus aureus* lineages in two public hospitals in Rio de Janeiro, Brazil. Braz. J. Infect. Dis..

[14] McCulloch JA (2015). Complete genome sequence of *Staphylococcus aureus* FCFHV36, a methicillin-resistant strain heterogeneously resistant to vancomycin. Genome Announc..

[15] Kuroda M (2001). Whole genome sequencing of meticillin-resistant *Staphylococcus aureus*. Lancet.

[16] Hiramatsu K (1997). Dissemination in Japanese hospitals of strains of *Staphylococcus aureus* heterogeneously resistant to vancomycin. Lancet.

[17] Coelho LR (2008). *agr* RNAIII divergently regulates glucose-induced biofilm formation in clinical isolates of *Staphylococcus aureus*. Microbiology.

[18] Gong J (2014). The accessory gene regulator (*agr*) controls *Staphylococcus aureus* virulence in a murine intracranial abscesses model. Braz. J. Infect. Dis..

[19] Pollitt EJG, West SA, Crusz SA, Burton-Chellew MN, Diggle SP (2014). Cooperation, quorum sensing, and evolution of virulence in *Staphylococcus aureus*. Infect. Immun..

[20] Ishihara K (2014). Methicillin-resistant *Staphylococcus aureus* carriage among veterinary staff and dogs in private veterinary clinics in Hokkaido, Japan. Microbiol. Immunol..

[21] Jang Y (2014). Characterization of methicillin-resistant *Staphylococcus* spp. isolated from dogs in Korea. Jpn. J. Vet. Res..

[22] Worthing KA (2017). Molecular characterization of methicillin-resistant *Staphylococcus aureus* isolated from Australian animals and veterinarians. Microb. Drug Resist..

[23] Vincze S (2014). Alarming proportions of methicillin-resistant *Staphylococcus aureus* (MRSA) in wound samples from companion animals, Germany 2010–2012. PLoS One.

[24] Iverson SA (2015). Anatomical patterns of colonization of pets with staphylococcal species in homes of people with methicillin-resistant *Staphylococcus aureus* (MRSA) skin or soft tissue infection (SSTI). Vet. Microbiol..

[25] Bes TM (2018). Prevalence of methicillin-resistant *Staphylococcus aureus* colonization in individuals from the community in the city of Sao Paulo, Brazil. Rev. Inst. Med. Trop. São Paulo..

[26] Ferreira JP (2011). Transmission of MRSA between companion animals and infected human patients presenting to outpatient medical care facilities. PLoS One.

[27] Vincze S (2014). Risk factors for MRSA infection in companion animals: Results from a case–control study within Germany. Int. J. Med. Microbiol..

[28] Loeffler A (2013). Whole-genome comparison of meticillin-resistant *Staphylococcus aureus* CC22 SCC*mec*IV from people and their in-contact pets. Vet. Dermatol..

[29] Davis MF (2015). Genome sequencing reveals strain dynamics of methicillin-resistant *Staphylococcus aureus* in the same household in the context of clinical disease in a person and a dog. Vet. Microbiol..

[30] Silva-Carvalho MC (2009). Emergence of multiresistant variants of the community-acquired methicillin-resistant *Staphylococcus aureus* lineage ST1-SCC*mec*IV in 2 hospitals in Rio de Janeiro, Brazil. Diagn. Microbiol. Infect. Dis..

[31] Martins A, Moraes Riboli DF, Cataneli Pereira V, de Lourdes Ribeiro de Souzada Cunha M (2014). Molecular characterization of methicillin-resistant *Staphylococcus aureus* isolated from a Brazilian university hospital. Braz. J. Infect. Dis..

[32] Silva-Carvalho MC (2009). Comparison of different methods for detecting methicillin resistance in MRSA isolates belonging to international lineages commonly isolated in the American continent. Microbiol. Immunol..

[33] de Sousa-Junior FC (2009). Genotyping of methicillin-resistant *Staphylococcus aureus* isolates obtained in the Northeast region of Brazil. Braz. J. Med. Biol. Res..

[34] Costa MOC (2013). Complete genome sequence of a variant of the methicillin-resistant *Staphylococcus aureus* ST239 lineage, strain BMB9393, displaying superior ability to accumulate *ica*-independent biofilm. Genome Announc..

[35] Teixeira MM (2012). Emergence of clonal complex 5 (CC5) methicillin-resistant *Staphylococcus aureus* (MRSA) isolates susceptible to trimethoprim-sulfamethoxazole in a Brazilian hospital. Braz. J. Med. Biol. Res..

[36] Soares Magalhães RJ (2010). Risk factors for methicillin-resistant *Staphylococcus aureus* (MRSA) infection in dogs and cats: A case–control study. Vet. Res..

[37] Challagundla L (2018). Phylogenomic classification and the evolution of clonal complex 5 methicillin-resistant *Staphylococcus aureus* in the western hemisphere. Front Microbiol..

[38] Harrison E, Weinert L (2014). A shared population of epidemic methicillin-resistant *Staphylococcus aureus* 15 circulates in humans and companion animals. MBio.

[39] Paterson GK (2015). Capturing the cloud of diversity reveals complexity and heterogeneity of MRSA carriage, infection and transmission. Nat. Commun..

[40] Richardson EJ (2018). Gene exchange drives the ecological success of a multi-host bacterial pathogen. Nat. Ecol. Evol..

[41] Lowder BV (2009). Recent human-to-poultry host jump, adaptation, and pandemic spread of *Staphylococcus aureus*. Proc. Natl. Acad. Sci. U.S.A..

[42] Guinane CM (2010). Evolutionary genomics of *Staphylococcus aureus* reveals insights into the origin and molecular basis of ruminant host adaptation. Genome Biol. Evol..

[43] Abbott Y, Leonard FC, Markey BK (2010). Detection of three distinct genetic lineages in methicillin-resistant *Staphylococcus aureus* (MRSA) isolates from animals and veterinary personnel. Epidemiol. Infect..

[44] Gómez-Sanz E, Torres C, Lozano C, Zarazaga M (2013). High diversity of *Staphylococcus aureus* and *Staphylococcus pseudintermedius* lineages and toxigenic traits in healthy pet-owning household members. Underestimating normal household contact?. Comp. Immunol. Microbiol. Infect. Dis..

[45] Howe RA, Monk A, Wootton M, Walsh TR, Enright MC (2004). Vancomycin susceptibility within methicillin-resistant *Staphylococcus aureus* lineages. Emerg. Infect. Dis..

[46] da Costa TM (2016). Clinical and microbiological characteristics of heteroresistant and vancomycin-intermediate *Staphylococcus aureus* from bloodstream infections in a Brazilian teaching hospital. PLoS One.

[47] Gardete S, Tomasz A (2014). Mechanisms of vancomycin resistance in *Staphylococcus aureus*. J. Clin. Invest..

[48] Sasaki T (2010). Multiplex-PCR method for species identification of coagulase-positive staphylococci. J. Clin. Microbiol..

[49] The Clinical and Laboratory Standards Institute (2016). Performance Standards for Antimicrobial Susceptibility Testing CLSI supplement M100S.

[50] Oliveira DC, de Lencastre H (2002). Multiplex PCR strategy for rapid identification of structural types and variants of the mec element in methicillin-resistant *Staphylococcus aureus*. Antimicrob. Agents Chemother..

[51] Boye K, Bartels MD, Andersen IS, Møller JA, Westh H (2007). A new multiplex PCR for easy screening of methicillin-resistant *Staphylococcus aureus* SCC*mec* types I-V. Clin. Microbiol. Infect..

[52] Cockfield JD, Pathak S, Edgeworth JD, Lindsay JA (2007). Rapid determination of hospital-acquired meticillin-resistant *Staphylococcus aureus* lineages. J. Med. Microbiol..

[53] Beltrame CO (2012). Restriction modification (RM) tests associated to additional molecular markers for screening prevalent MRSA clones in Brazil. Eur. J. Clin. Microbiol. Infect. Dis..

[54] Teixeira LA (1995). Geographic spread of epidemic multiresistant *Staphylococcus aureus* clone in Brazil. J. Clin. Microbiol..

[55] Enright MC, Day NP, Davies CE, Peacock SJ, Spratt BG (2000). Multilocus sequence typing for characterization of methicillin-resistant and methicillin-susceptible clones of *Staphylococcus aureus*. J. Clin. Microbiol..

[56] von Eiff C, Friedrich AW, Peters G, Becker K (2004). Prevalence of genes encoding for members of the staphylococcal leukotoxin family among clinical isolates of *Staphylococcus aureus*. Diagn. Microbiol. Infect. Dis..

[57] Zerbino DR (2010). Using the Velvet de novo assembler for short-read sequencing technologies. Curr. Protoc. Bioinform..

[58] Kearse M (2012). Geneious Basic: An integrated and extendable desktop software platform for the organization and analysis of sequence data. Bioinformatics.

[59] Angiuoli SV (2008). Toward an online repository of standard operating procedures (SOPs) for (meta)genomic annotation. OMICS.

[60] Bertels F, Silander OK, Pachkov M, Rainey PB, van Nimwegen E (2014). Automated reconstruction of whole-genome phylogenies from short-sequence reads. Mol. Biol. Evol..

[61] Didelot X, Wilson DJ (2015). ClonalFrameML: Efficient inference of recombination in whole bacterial genomes. PLoS Comput. Biol..

[62] Suchard MA (2018). Bayesian phylogenetic and phylodynamic data integration using BEAST 1.10. Virus Evol..

[63] Tavaré. Some probabilistic and statistical problems in the analysis of DNA sequences. in *Lectures on Mathematics in the Life Sciences.* (ed. M., M. R.) 57–86 (1985).

[64] Yang Z (1994). Maximum likelihood phylogenetic estimation from DNA sequences with variable rates over sites: Approximate methods. J. Mol. Evol..

[65] Drummond AJ, Ho SYW, Phillips MJ, Rambaut A (2006). Relaxed phylogenetics and dating with confidence. PLoS Biol..

[66] Kingman JFC (1982). The coalescent. Stoch. Process. Appl..

[67] Duchêne S (2016). Genome-scale rates of evolutionary change in bacteria. Microb. Genom..

[68] Rambaut, A. G. & Drummond, A. J. Tracer. http://beast.bio.ed.ac.uk/Tracer (2013).

[69] Rambaut, A. & Drummond, A. J. TreeAnnotator. https://beast.community/treeannotator (2013).

[70] Rambaut, A. FigTree. http://tree.bio.ed.ac.uk/software/figtree (2007).

[71] Alikhan N-F, Petty NK, Ben Zakour NL, Beatson SA (2011). BLAST ring image generator (BRIG): Simple prokaryote genome comparisons. BMC Genom..

[72] Darling AE, Mau B, Perna NT (2010). ProgressiveMauve: Multiple genome alignm. PLoS One.

[73] Arndt D (2016). PHASTER: A better, faster version of the PHAST phage search tool. Nucleic Acids Res..

[74] Zankari E (2017). PointFinder: A novel web tool for WGS-based detection of antimicrobial resistance associated with chromosomal point mutations in bacterial pathogens. J. Antimicrob. Chemother..

[75] Bortolaia V (2020). ResFinder 4.0 for predictions of phenotypes from genotypes. J. Antimicrob. Chemother..

[76] Langille MGI, Brinkman FSL (2009). IslandViewer: An integrated interface for computational identification and visualization of genomic islands. Bioinformatics.

[77] Camacho C (2009). BLAST+: Architecture and applications. BMC Bioinform..

[78] Irazoqui JE, Urbach JM, Ausubel FM (2010). Evolution of host innate defence: Insights from *Caenorhabditis elegans* and primitive invertebrates. Nat. Rev. Immunol..

[79] Sifri CD, Begun J, Ausubel FM, Calderwood SB (2003). *Caenorhabditis elegans* as a model host for *Staphylococcus aureus* pathogenesis. Infect. Immun..

