# Supplementary figures and images for: Comparative genomics of MRSA strains from human and canine origins reveals similar virulence gene repertoire
Source: Sci Rep. 2021 Feb 25;11:4724. doi: 10.1038/s41598-021-83993-5 (PMC7907190; doi:10.1038/s41598-021-83993-5)

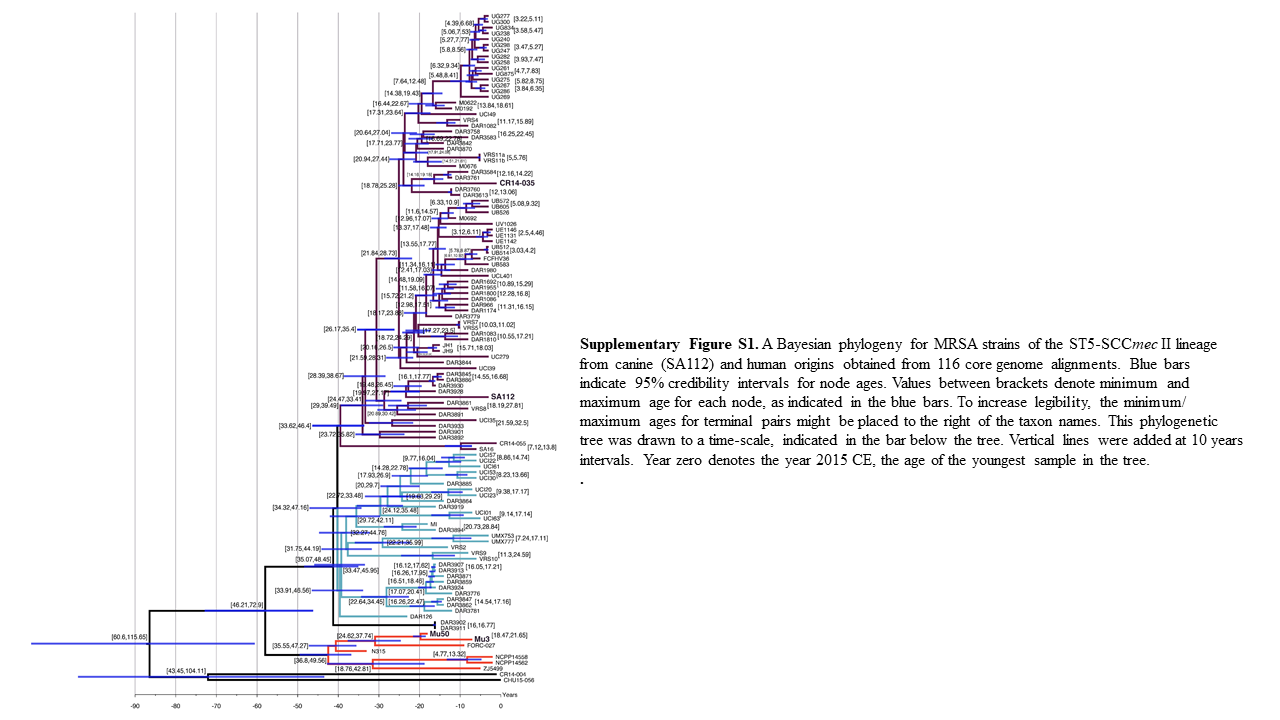

Supplement: Supplementary file 1 — Supplementary figure S1. [file 41598_2021_83993_MOESM1_ESM.tif]
